# A Mixed-method Study on the Implementation of a Medical Psychiatric Care Unit

**DOI:** 10.5334/ijic.8964

**Published:** 2025-07-28

**Authors:** Chedwa Pinto, Maaike Meeder, Jan Busschbach, Witte Hoogendijk, Jelmer Alsma, Isabelle Fabbricotti, Maarten van Schijndel

**Affiliations:** 1 Department of Psychiatry, Erasmus University Medical Center, Rotterdam, the Netherlands; 2 Department of Medical Psycholog, Erasmus University Medical Center, Rotterdam, the Netherlands; 3 Department of Internal Medicine, Erasmus University Medical Center, Rotterdam, the Netherlands; 4 Erasmus School of Health Policy and Management, Erasmus University Rotterdam, Rotterdam, the Netherlands

**Keywords:** integrated care, medical psychiatry units, implementation, context, evidence-based practice

## Abstract

**Introduction:**

Medical Psychiatry Units (MPUs) offer integrated care for patients needing both medical and mental healthcare in hospitals. MPUs provide unique care not covered by Psychiatric Consultation-Liaison Services (PCS). Despite their increasing number, little research exists on why referrers choose MPUs, though their perspectives shape patient populations and reflect MPU goals.

**Aims:**

**1.** Asses physician satisfaction with the Erasmus Medical Center MPU and its alignment with initial expectations.
**2a.** Evaluate whether referrals match the intended patient population needing disruptive behavior management and compare this with PCS referrals.
**2b.** Identify unintended reasons for MPU referrals.

**Method:**

We conducted interviews with physicians and analyzed questionnaires on MPU or PCS referrals from November 2020 to May 2021.

**Results:**

**1.** Physicians report that the MPU improves care quality, job satisfaction, collaboration, safety, educational value and reduces stigmatization.
**2a.** Among 62 referrals, the MPU admitted nearly twice the disruptive patients.
**2b.** Common MPU referral reasons included nursing care load, department expertise and suicidality, while PCS referrals were more often due to delirium.

**Conclusion:**

The Erasmus MC MPU meets its founding goals. Physicians recognize its added value, particularly in managing patients with disruptive behaviors and high care needs. The integration of medical and psychiatric expertise has fulfilled expectations.

## Introduction

The presence of psychiatric problems among medical inpatients is linked to increased healthcare utilization and costs. Therefore, hospitals are turning to Medical Psychiatry Units (MPUs) to provide integrated medical- and psychiatric healthcare. MPUs are categorized into four types based on their medical and psychiatric capabilities. Despite their existence since the 1980s, MPUs are not widely available globally. MPUs provide care in hospitals not already offered by Psychiatric Consultation- and Liaison Services (PCS) [1].

The literature details 12 academic MPUs, with 6 of them sharing a design similar to the Erasmus MC MPU, meaning units offering combined medical and psychiatric inpatient treatment, under medical auspices (the medical/psychiatric model) [2,3,4,5,6,7,8,9]. These MPUs, situated in university hospitals, cater to adult populations and are overseen by a medical physician and an attending psychiatrist. Despite their comparable designs, these MPUs serve different populations and have distinct objectives. Some MPUs focus more on psychiatric populations, while others are geared towards medical populations. While they generally treat similar issues such as mood disorders, agitation, delirium or psychosis, the specific behavioral focus can vary. For example, some MPUs address issues like uncooperativeness [7], while others focus on treating suicidal patients, managing wandering behaviors, or handling aggression [5,9]. Furthermore, some MPUs specialize in long-term care for patients with somatoform disorders [6]. Even within this specific group of studies, the exact descriptions of the goals and populations often lack clarity and directness.

Typically, research focuses on patients already admitted to MPUs, overlooking the referrer’s perspectives on why patients should be admitted, while this referrer’s perspective is relevant as it shapes the MPU population. One study by Caarls et al. did examine referrers’ views through a concept mapping study on physicians’ and psychiatrists’ opinion on which populations are eligible for MPU admission. However, this was not a real-life study of referral reasons to MPU or PCS [10]. The diversity in MPU contexts, goals, designs, and patient populations poses challenges when employing evidence-based medicine methodologies, as it is difficult to generalize results from one MPU to another. Moreover, because MPUs are part of an integrated care chain, often involving patients and doctors in stressful and sometimes involuntary circumstances, controlled study designs requiring informed consent are complex [11]. Consequently, despite over four decades of MPU development, substantive evidence regarding their effectiveness still needs to be provided. In an alternative approach to assess MPU effectiveness, we put emphasis on physicians’ referrals and experiences to gain insights into the goals and achievements of an MPU. By exploring their expectations and experiences with the MPU of the Erasmus MC, we aim to endorse the evidence-based practice (EBP) of MPUs as an integrated care approach for medical patients with psychiatric problems.

### Implementation context

Erasmus MC University Medical Center in Rotterdam, the Netherlands, is a tertiary academic hospital serving as a supraregional center for acute, complex, and rare disease cases. It has 1,125 beds and a staff of 14,000. The Department of Internal Medicine houses 38 beds, including 7 in the Medical Psychiatry Unit (MPU). The Department of Psychiatry, a 15-minute walk away, has 45 beds and focuses on severe depression, first psychosis, and psychiatric care during pregnancy. The psychiatric consultation service (PCS) includes four psychiatrists, four clinical nurse specialists, and rotating psychiatry and general practice residents.

The history of the MPU, which began in 2011, was reconstructed using documents from the steering committee and working group, along with interviews with key stakeholders. In 2013, a steering committee—composed of leaders from Internal Medicine, Psychiatry, Gastrointestinal Diseases, and Neurology—took on the mission of supporting patients with medical and psychiatric comorbidities. Development was later delegated to a dedicated working group of specialists, nurses, and support staff. In 2018, MPU nurse training began, aligned with the hospital’s reconstruction. The MPU officially launched in 2019 alongside the new hospital, aiming to improve care for patients who do not fit into conventional medical or psychiatric wards. Its overarching goals were to enhance care quality and safety while reducing costs through integrated care.

Located on the same floor as geriatrics, the MPU includes suicide prevention measures such as lockable doors and camera surveillance. It is co-attended by a dedicated internist and two PCS psychiatrists. Though initially linked to Internal Medicine, the MPU now accepts referrals from other specialties. Patients are jointly triaged by the internist and psychiatrist to ensure appropriate admission, excluding those with low medical or behavioral acuity.

It took several months to implement the current care model. Each specialty is expected to attend morning rounds; psychiatrists and internists attend together, separate from other specialists. Referring physicians are consulted as needed, either in person or by phone. Some collaborations, like physiotherapy, remain informal. Psychiatric aftercare is organized by the psychiatry aftercare office, and weekly multidisciplinary meetings support coordinated care.

According to internal guidelines, the MPU admits patients requiring medical treatment along with behavioral disorders that interfere with care on regular wards. Most admissions are unplanned, including suicide attempts, patients needing safety measures, or those requiring further psychiatric assessment.

Nurse training for the MPU has led many Internal Medicine nurses to become dedicated MPU staff. A psychiatric nurse specialist also works alongside psychiatrists and the internist. Despite their primary medical background, MPU nurses maintain a therapeutic environment. The MPU’s reputation positively influences nursing recruitment.

In conclusion, an MPU has been successfully established at Erasmus MC, meeting structural and operational goals. Whether it fully meets its intended mission remains a relevant question: if not, one can question its rationale. Therefor this article aims to explore whether the MPU at ethe Erasmus MC lives up to the original expectations.

### Aims

The present study aims to address the following questions:

**1.** How do physicians, as primary stakeholders, value the MPU? Is this in line with the initial expectations and intentions?
**2a** Do referrals match the intended patient population of patients requiring medical treatment combined with a behavioral disorder that disrupts treatment on the regular ward, and how does this population differ from the PCS population?
**2b** Are there unintended patients groups present, like patients with somatoform disorders and geriatric patients with dementia and wandering behaviors? Are there any unintended reasons for referral, such as bed shortages, that are frequently practiced? If such unintended reasons for referral exist, do they differ from referrals to the PCS? Are these unintended reasons for referrals undesirable?

## Theory and Methods

### Theoretical framework

Evidence-based practice (EBP) is a comprehensive approach that considers four types of evidence: scientific, experiential, stakeholder, and organizational. In our current EBP approach, we focus on the latter two as we examine the initial intentions and resulting organizational structure of the MPU. We investigate whether the Erasmus MC MPU fulfills the original intentions outlined in its founding documentation. We have conducted interviews with physicians and analyzed current referrals to the Erasmus MC MPU to gain insight into their perspectives.

### Methods

This preliminary assessment uses different methods to gather information about the potential variance between the plans and the actual implementation of the Erasmus MC MPU from physicians’ perspectives. This investigation ran from November 2020 to May 2021. The methods are listed below. We received ethical approval for the study design and procedures from the Erasmus MC ethics review board (METC number 2023-0241). When interviewing stakeholders, they provided informed consent by phone and confirmed it again before the interviews.

#### First aim: Physicians opinions on the value of the MPU

To achieve our first aim, we conducted semi-structured interviews with physicians about their views on the added value of the Erasmus MC MPU. The interviews covered topics such as improving the quality of care, patient safety (especially for patients with suicidal behavior), and the potential outcomes of the MPU if a hypothetical cost-benefit analysis would be done (see the appendix for the topic list). Interviewees were chosen based on their specialties to ensure a balanced representation of those who regularly refer patients to the MPU and those who do so infrequently or never. The interview topics were predefined by the psychiatrists working at the MPU, and interviews continued until data saturation was reached.

#### Second aim: actual referrals vs. the intended patient population

For our second aim, comparing actual referrals to the intended patient population, we conducted a consecutive sampling survey of referral reasons to the Erasmus MC MPU and PCS by examining patients’ medical records. We obtained informed consent from patients via email, and non-responses were considered as implied consent in line with national regulations. Referrals from psychiatric departments were excluded to prevent potential bias in the selection and evaluation of MPU referrals.

##### Demographics and diagnoses

We gathered patient demographics as well as medical and psychiatric diagnoses from electronic medical records.

##### Telephone interviews

Telephone interviews were conducted with referring physicians to the MPU and PCS within 24 hours of referral, or two to three days if the referral occurred over the weekend. Referral reasons were assessed using two questionnaires: an “Ad Hoc Questionnaire” and the validated “Inpatient Disruptive Behavior Index,” which will be detailed below. The results were then compared against the MPU’s inclusion criteria.

##### Inpatient Disruptive Behavior Index

The Inpatient Disruptive Behavior Index (InDiBI, see Appendix) is a simplified observational tool used to identify disruptive behaviors and evaluate their manageability. This tool consists of a general question about patient manageability, scored on a three-level scale, and a second question that enables the rating of various disruptive behaviors. It is expected that patients referred to the MPU will exhibit a higher frequency of unmanageable behavior and demonstrate a broader range of disruptive behaviors compared to the PCS.

##### Ad Hoc Questionnaire

We used an Ad Hoc Questionnaire (AHQ, see Appendix) to determine whether the inclusion criteria for the Erasmus MC MPU were indeed reasons for referral and to explore additional referral reasons. This questionnaire structure is based on Caarls’ cluster model, which classifies determinants for MPU admission into five categories: 1. Staff competencies and organizational prerequisites, 2. Patient characteristics, 3. Psychiatric symptoms and behavioral problems, 4. Medical needs and capabilities, and 5. Patient context [12]. Questions related to categories 2 to 5 were adapted to fit the study’s context, with modifications reviewed by medical staff to ensure completeness and relevance.

#### Data analysis

In this research design, both quantitative and qualitative methods were used, making it descriptive and exploratory. Because of the exploratory nature of the study, a power calculation is less relevant, allowing for an ad hoc analysis of the collected data.

#### Qualitative Analysis of Interview Data

For the first aim, some of the qualitative data derived from responses to open-ended questions underwent content analysis. Values statements from these responses were categorized into broader value themes, with some responses fitting into predetermined themes. The frequency of each theme per informant was counted and interpreted in light of specialist perspectives. The interviews with participants’ consent were recorded, transcribed, and organized into themes and subthemes. Consensus meetings among the three researchers were held to discuss the transcriptions.

#### Quantitative analysis of questionnaire data

For the second aim, the study compared the Erasmus MC MPU and PCS groups based on two parameters: 1) manageability as assessed with the InDiBI, including the overall count of disruptive behaviors and the quantity of specific disruptive behaviors, and 2) the number of referral reasons for each category, as evaluated in the AHQ. Chi-square tests were conducted for analysis of single item questions, two sided independent samples t-test of means for question clusters. Given that the dependent variables, i.e. the number of positive answers on the AHQ and the InDiBi, are inherently not normally distributed, the median and interquartile range (IQR) of the outcomes were also examined, in addition to the mean and standard deviation (Table 3).

## Results

### First aim: Physicians’ opinions on the MPU’s Added Value

Ten physicians, four psychiatrists, and six medical practitioners from various specialties participated in the qualitative interviews (Table 1). Their consensus was that the Erasmus MC MPU provides significant value beyond the PCS, entailing improved quality of care, increased job satisfaction, better cross-discipline collaboration, and enhanced patient and staff safety measures. Importantly, psychiatrists emphasized the role of the Erasmus MC MPU in reducing stigmatization, while the other medical specialists highlighted the MPU’s value as an educational environment.

**Table 1:** Interview themes per participating physicians.


DISCUSSED ITEM	QUALITY OF CARE	JOB SATISFACTION	SAFETY	COLLABORATION	STIGMATIZATION	LEARNING ENVIRONMENT

Psychiatrist 1	1	0	1	1	1	0

Psychiatrist 2	1	1	0	1	0	0

Psychiatrist 3	1	1	0	1	1	0

Psychiatrist 4	1	0	1	1	1	1

Vascular Surgeon	1	1	0	1	0	0

Oncology surgeon	1	0	1	1	0	0

Intensivist	1	1	0	1	0	1

Internist	1	1	1	1	0	1

Endocrinologist	1	0	1	1	0	0

Trauma surgeon	1	1	1	0	0	0

Total	10	6	6	9	3	3


#### Quality of Care

All ten physicians noted a significant improvement in the quality of care. They unanimously praised the multidisciplinary approach, which they believed led to enhanced patient treatment plans. For example, they mentioned how the expertise of the MPU personnel helped in the early identification of specific symptoms, such as the onset of mania in bipolar disorder, thereby preventing behavioral issues. Other protective factors included maintaining a serene ward environment and adopting individualized care approaches.

Some physicians suggested that the MPU ensures care continuity due to the constant presence of two psychiatrists as co-attending physicians. They contrasted this with the PCS, where short consultations and weaker adherence to advice disrupt continuity. One physician noted that the Erasmus MC MPU “psychiatrists are familiar with the patients and their specific issues.” Consequently, the MPU discusses psychiatric issues in greater depth than traditional nursing wards. Physicians also remarked on the improved attention to patients who have attempted suicide and the ability to oversee complex medical patient care, partly because the MPU eliminates the need for back-and-forth ward referrals for different care needs. One physician commented: “There is no better place for these patients”.

Lastly, improvements in patient transfers from the Erasmus MC were reported, due to constant multidisciplinary cooperation between physicians, the admission coordinator, and the aftercare office. Physicians recognized that merging knowledge between the medical and psychiatric staff was crucial for this improvement.

Some physicians expressed concerns that the MPU might be associated with higher costs, citing factors such as prolonged lengths of stay and an elevated nurse-to-bed ratio. Despite these concerns, there was unanimous agreement that it represents the highest standard of care for patients with complex behavioral health conditions. They emphasized the advantages of improved continuity of care and a decreased rate of readmissions. These factors could potentially lead to lower long-term costs. One physician stressed the idea that “investing in quality is worthwhile”.

The interviews revealed several misconceptions about the Medical Psychiatric Unit (MPU). For example, some physicians incorrectly assumed that the MPU was consistently operating at full capacity, despite being unable to provide specific examples of rejected referrals. One physician admitted to never having made a referral to the MPU, while another thought that the MPU nursing staff required significant guidance in providing medical care. However, in practice, the nursing team predominantly consists of professionals with a strong medical background.

#### Patient and Personnel Safety

Six physicians from different specialties, including surgeons and psychiatrists, agreed that the MPU significantly enhances patient and staff safety. Locked doors prevent MPU patients from unsupervised wandering, camera surveillance ensures better monitoring of suicide attempts by patients, and hospital security responds more promptly and effectively when needed at the MPU. Camera surveillance also reduces the need for patient restraints and allows for quicker de-escalation of such measures. They suggested that PCS-treated patients might require more frequent and prolonged restraint than MPU patients.

Special procedures, like patient restraints, require meticulous documentation. One doctor pointed out that legal knowledge and other skills to perform such meticulous documentation is often lacking among many doctors and nursing staff, whereas the MPU team possesses adequate legal expertise and other necessary skills. One physician erroneously suggested that the locked doors and camera surveillance at the MPU provided better control over ‘prisoners’, even though the MPU does not cater to this population.

#### Job Satisfaction

Six physicians from various specialties, including a psychiatrist, a vascular surgeon, an internist and an intensivist, reported increased job satisfaction at both the MPU and the regular nursing wards. They noted that the presence of experienced nursing staff reduces stress, as the MPU is responsible for these complex patients.

#### Inter-specialty collaboration

A majority of nine physicians agreed that inter-specialty collaboration had improved. Some physicians mentioned the considerable geographical distance of the MPU from their ‘own’ nursing wards. Nevertheless, most physicians reported fewer complications in patient redirection between specialties since the MPU’s establishment. One doctor recalled that communication was sometimes sporadic and inadequate when a medical problem ceased to be acute while behavioral issues persisted. Since the MPU’s establishment, this issue has become less common due to maintaining regular contact with the referring physician. Furthermore, one doctor cited the value of the MPU’s psychiatric nursing expertise: “[…] I believe the opinion of the nurses is essential; it’s better to have an MPU nurse because they have an informed perspective on psychiatric patients […].”

#### Reducing stigmatization

Three psychiatrists commented on the reduction of stigmatization toward psychiatry. Some regular nursing wards would resort to stereotypical language when discussing psychiatric patients. They viewed the psychiatric department as resistant in an otherwise medically focused hospital. The introduction of the MPU has reduced this perceived rift.

#### The Erasmus MC MPU as a Learning Environment

Aligned with the themes above, three physicians, none of whom were psychiatrists, discussed the MPU as an educational platform. The specialized department enables residents to manage complex patients instead of having infrequent encounters, allowing them to learn how to treat this patient group optimally. Established physicians also benefit from collaborative learning opportunities with their colleagues at the MPU.

### Second aim: Patient population served by the MPU

From November 2020 to May 2021 there were 96 referrals. Of these, we included 67 patients and excluded 29 because we could not reach their referring physician (MPU n = 12, PCS n = 17). Of these, 62 patients consented: 31 referrals to the MPU and an equal number to the PCS. Table 1 illustrates that the PCS had a higher representation of male, neurosurgery, hematology, COVID-19, and delirium patients. In contrast, the MPU had a larger number of surgery, intoxication, and psychosis patients.

#### Do referrals match the intended patient population?

To address the first part of the second study aim, which examines whether referrals match the intended population of patients needing medical treatment combined with a behavioral disorder that disrupts regular ward treatment:

- Table 3 shows that although the MPU had twice as many patients categorized as “unmanageable”(8 versus 4), the majority were still perceived as manageable.
- MPU patients had a higher overall count of disruptive behavioral symptoms, with a median of 4 compared to 2 in PCS.
- Delusions showed the most (significant) disparity in types of disruptive behavior, with 14 MPU patients versus 3 in PCS (45% vs 10%), followed by self-harm: 8 versus 1 (26% vs 3%). Several behaviors, like wandering, are similar between the two groups.

#### Are there any unintended patient populations or reasons for referral to the MPU?

To address the second part of the second study aim:

- Table 2 shows an equal, small number of patients with somatoform disorders in both MPU and PCS, as well as a small number of patients with neurocognitive disorders. The mean age of MPU patients is lower than that of PCS patients.
- Table 3 highlights the most significant differences between MPU and PCS, and several behaviors, like wandering, that are similar between the two groups.
- Tables 4 and 5 present significant differences in the Staff Competencies and Patient Characteristics clusters. In the Staff Competencies cluster, the items ‘the nursing care load is too high’ (21 vs. 8) and ‘there is insufficient expertise on the current department’ (24 vs. 12) showed notable differences between MPU and PCS.

**Table 2:** Patient characteristics, referring specialties and diagnoses, November 2020 to May2021.


CHARACTERISTICS	MPU N = 31	PCS N = 31

Male	54.8%	74.2%

Mean Age in years (range)	49.52 (24–83)	54.35 (19–74)

Referring specialties		

(Acute) internal medicine	8	6

Neurology	5	2

Surgery	5	0

Cardiology	3	4

Neurosurgery	2	6

Nephrology	1	0

Otorhinolaryngology	1	0

Pulmonology	1	4

Dermatology	1	0

Oncology	1	2

Endocrinology	1	0

Obstetrics and Gynecology	1	1

Geriatrics	1	0

Hematology	0	3

Plastic surgery	0	1

Urology	0	1

Gastro-enterology	0	1

Medical diagnosis		

Intoxication	5	0

Neoplasms	3	5

Traumatic wounds	3	0

Neurotrauma/hemorrhage/angiopathy	3	2

Cardiac arrest/infarction/failure	3	4

Auto-immune (encephalitis/SLE/myositis)	2	2

Epilepsy	1	0

Physical decline/electrolyte disorder	2	0

Pneumococcal pneumonia	1	0

COVID-19	1	7

Kidney transplant	1	0

Acute renal failure	1	0

Hydrocephalus	0	2

Myelodysplasia	0	2

Bilateral lung transplant	0	1

Porphyria	0	1

Miscellaneous	5	5

Psychiatric diagnosis		

Schizophrenia / psychotic disorders	9	3

Delirium	5	20

Depressive disorder	5	2

Disorders due to substance use/intoxication or addictive behaviors	4	0

Bipolar or related disorders	2	0

Neurocognitive disorders (excluding delirium)	2	0

Anxiety or fear-related disorders	1	4

Conversion disorder	1	1

Psychogenic Non-Epileptic Disorder	1	0

Personality Disorder	1	1


**Table 3:** InDiBI Scores for 31 MPU and 31 PCS Patients.


DEPARTMENT	MPU	PCS

**Perception of Manageability**		

Number of manageable patients	23	27

**Number of Disruptive Behaviors (max = 14 per patient)**		

Total	112	62

Minimum	1	0

Maximum	8	6

Range	7	6

Median	4	2

IQR	2–5	1–3

**Number of cases per behavior**		

Aggression	6	2

Suicidal behavior*	9	3

Self-harm*	8	1

Claiming behavior	3	5

Uncooperative	14	11

Delusions*	14	3

Hallucinations	6	5

Agitation*	19	11

Disinhibition or loss of decorum	7	2

Shouting and screaming	9	4

Wandering	4	3

Inertia	3	2

Apathy	7	9

Refusal to eat and/or drink	3	1


*P ≤ 0.05: on chi-square test.

**Table 4:** Referral reasons according to the Ad hoc questionnaire.


	N	MEAN	SD	MEDIAN	IQR	P t-test	TIMES “YES”

	1: Staff Competencies: 7 items						

MPU	31	3.35	1.33	3	2	<0.001	104 (out of 217)
	
PCS	31	1.74	1.29	2	1		54

	2: Patient Characteristics: 8 items						

MPU	31	4.97	1.42	5	2	0.007	154 (out of 248)
	
PCS	31	4.00	1.32	4	2		124

	3: Psychiatric symptoms and Behavioral problems: 6 items						

MPU	31	1.70	1.32	2	1	0.45	53 (out of 186)
	
PCS	31	1.09	1.01	1	2		34

	4: Medical needs and capabilities: 1 item						

MPU	31	0.29	0.46	0	1	0.22	9 (out of 31)
	
PCS	31	0.16	0.37	0	0		5

	5: Patient context: 1 item						

MPU	31	0.35	0.49	0	1	0.40	11 (out of 31)
	
PCS	31	0.26	0.44	0	1		8


**Table 5:** Number of ‘yes’ answers per item of the Ad hoc questionnaire.


QUESTION		MPU	PCS

	*Question 1–7 : Staff competencies [Caarls’ Cluster 1]*	*‘Yes’*	*‘Yes’*

1	Preventive, to prevent behavioral and/or psychological problems	10	10

2	There is insufficient expertise on the current department*	24	12

3	The nursing care load is too high because of the psychiatric problems*	21	8

4	Aftercare is needed for the psychiatric problem*	20	12

5	There are problems surrounding the patient’s discharge (not being psychiatric aftercare)*	8	1

6	There is a shortage of beds on the regular ward	4	2

7	The problem is too complicated for treatment elsewhere*	17	9

	*Question 8–15: Patient characteristics [ Caarls’ Cluster 2]*		

8	A psychiatric problem is prominent*	27	9

9	Further diagnostics are needed to determine the cause of the behavior (psychological or medical)	9	9

10	There is a psychiatric history*	25	14

11	There is a history of substance abuse	13	6

12	A psychiatric or behavioral disorder interferes with the medical treatment	17	20

13	Treatment on the regular ward is impeded by the psychiatric or behavioral problem or vice versa*	22	14

14	Sufficient recovery is expected within the foreseeable future for transfer to a psychiatric or medical ward, elsewhere or home*	31	25

15	The psychiatric or behavioral problem is the result of a medical treatment or medical condition*	10	27

	*Question 16–21: Psychiatric symptoms and Behavioral problems [Caarls’Cluster 3]*		

16	There is a personality disorder	8	5

17	There is an intoxication or substance abuse issue	11	5

18	Delirium is present*	8	16

19	The patient has committed a suicide attempt*	10	1

20	There is evidence of suicidality*	10	2

21	There is aggression towards others	6	5

	*Question 22: Medical needs and Capabilities [Caarls’ Cluster 4]*		

22	There is a problematic interaction between patient and the medical staff	11	8

	*Question 23: Patient Context [Caarls’ Cluster 5]*		

23	There is a too complicated medical problem in a psychiatric patient	9	5


*P ≤ 0.05: on chi-square test.

Within the Patient Characteristics cluster, the most substantial differences were observed in ‘a psychiatric problem is prominent’ (27 vs. 9) and ‘the psychiatric or behavioral problem is the result of a medical treatment or condition’ (10 vs. 27).

Less substantial differences were noted in ‘treatment on the regular ward is impeded by the psychiatric or behavioral problem or vice versa’ (22 vs. 14) and ‘a psychiatric or behavioral disorder interferes with medical treatment’ (17 vs. 20). Both of these items align with the original aims of the MPU.

Within the Psychiatric symptoms and Behavioral problems cluster, the most significant differences were found in ‘delirium’ (8 vs. 16), ‘the patient has committed a suicide attempt’ (10 vs. 1), and ‘there is evidence of suicidality’ (10 vs. 2).

## Discussion

The literature reveals a diversity of MPU designs and populations. This underscores the need for comprehensive descriptions of MPU goals, designs, populations and outcomes. Following this, it is critical to assess whether these populations and goals are effectively addressed in real-world settings. Our study aimed to fill these gaps in understanding the effectiveness of MPUs by exploring how stakeholders’ perceptions shaped the establishment and operation of the Erasmus MC MPU and how well the current MPU lives up to their initial expectations. Furthermore, we investigated whether referrals match the original intended population.

### Stakeholders perceptions on Erasmus MC MPU’s value

When initiating the MPU at the Erasmus MC, the overarching goals were to enhance care quality and safety while reducing costs for patients with complex psychiatric and medical needs whom regular medical or psychiatric wards do not serve adequately. Regarding the first study aim, “How do physicians value the MPU?” physicians agree that the Erasmus MC MPU enhances care quality and safety. Particularly for suicidal patients, where better surveillance and a therapeutic environment help reduce self-harm behaviors. Physicians believe that enhanced treatment plans facilitate early identification and prevention of behavioral issues.

Physicians have identified an unplanned benefit: improved facilitation of patient transfers to inpatient and outpatient care. Physicians unanimously agree that the Erasmus MC MPU delivers high quality care for patients with complex behavioral issues, potentially leading to a reduced rate of readmissions. The MPU’s commitment to safety is evident in its measures, such as locked doors to prevent unsupervised wandering and reduce the need for patient restraints. The MPU’s focus on better monitoring of suicide attempters and quicker responses from hospital security has also been commended.

While some physicians noted the considerable geographical distance from their nursing wards, the majority reported increased job satisfaction in both the MPU and regular nursing wards. They also observed a decrease in complications during patient redirection between specialties. Psychiatrists have also praised the MPU for its role in reducing stigma and bridging the perceived gap between psychiatry and the rest of the hospital.

Other specialists value the Erasmus MC MPU as an educational environment.

Physicians who referred patients to the MPU less frequently appeared to have a less comprehensive understanding of its function, exemplified by the perception that the MPU is particularly suited for managing individuals described as “prisoners.”

### Patient population referred to the Erasmus MC MPU

Contrary to initial concerns, the MPU has not drawn long-stay somatoform cases, geriatric patients with dementia and wandering behaviors, or a surge of suicidal patients. Geriatric admissions remain low, likely reflecting Erasmus MC’s shift toward tertiary care. The MPU has sufficient capacity for unplanned admissions, such as suicide attempters, those requiring safety measures, or further diagnostic evaluation—patients who align with the defined target population.

In practice, inclusion criteria correspond well with the first part of the second study aim: “Do referrals match the intended patient population of patients with behavioral disorders that disrupt treatment on regular wards?” MPU patients display more frequent disruptive behaviors and suicidal tendencies compared to those referred to PCS. However, no significant differences were found between MPU and PCS in terms of patients considered ‘out of control,’ aggressive, or whose psychiatric or behavioral symptoms interfere with medical treatment.

Responses to the Ad Hoc questionnaire—where “high nursing care load” and “insufficient expertise” were common reasons for referral—suggest that patients are not necessarily unmanageable but that referrers feel unable to deliver optimal care. This aligns with semi-structured interview feedback, where all physicians emphasized the MPU’s added value in improving care quality.

Regarding the second part of the second study aim, findings indicate no unintended patient groups are being admitted. This is likely due to the careful triaging process, which ensures that patients with low medical or behavioral acuity are not admitted to the MPU. Referring ward staff often cite their own limitations or lack of psychiatric expertise as key reasons for referral, which may contribute to a high care burden. It is plausible that a staff member may manage one psychiatric issue well but struggle with another, making a patient manageable in one ward but not another. Because MPU staff are trained in psychiatric care, referrals for these reasons can still be appropriate and valuable.

Analysis showed that MPU referrals often involve delusions, agitation, self-harm, and suicidality, suggesting that the frequency and type of behavioral symptoms—rather than diagnosis—drive referrals. This trend applies to both MPU and PCS. However, MPU referrals more often cite psychiatric issues as the primary concern, with “a psychiatric problem in the foreground” noted three times more often than in PCS referrals. Conversely, PCS referrals more frequently involve delirium, cited twice as often. These patterns suggest staff refer to the MPU when behavioral symptoms dominate and to PCS when acute medical illness, particularly delirium, is the central issue.

### Literature contextualization

The Erasmus MC MPU integrates medical and psychiatric care within a structured environment, prioritizing the facilitation of medical treatment. This aligns with broader MPU-goals, such as managing disruptive behavior and improving patient-related and economic outcomes [13]. MPUs contribute to hospital functioning by addressing complex psychiatric cases, enhancing staff well-being in other departments, and improving access to care for these patients [11]. As highlighted in the introduction, the goals and target populations of MPUs are often ambiguous and lack clear definition. This study aimed to elucidate the objectives of the Erasmus MC MPU and identify the specific populations it serves. Our findings indicate that the objectives of the Erasmus MC MPU extend beyond merely facilitating medical treatment. They also include alleviating the burden on staff by providing care for patients whom they feel ill-equipped to manage, thereby promoting staff well-being.

While MPU objectives align with the Quadruple Aim—enhancing the care experience, improving provider well-being, and optimizing patient outcomes—they may compete, requiring careful balancing [14]. For example, patients and families may prioritize care continuity and longer hospital stays, providers may focus on safety and reducing staff strain, while payers emphasize cost containment. Unlike the Quadruple Aim’s focus on reducing population-level costs, MPUs impact hospital-related expenses [15,16].

Previous studies on MPUs have indeed largely focused on insurance-driven outcomes, such as reduced hospital Length of Stay (LOS) as a proxy for cost-effectiveness [13,16]. This study seeks to address this gap by evaluating broader MPU objectives at Erasmus MC, emphasizing patient experience, provider work-life, and patient outcomes. However, this evaluation is limited to the physicians’ perspective and excludes cost considerations.

When comparing the design of the Erasmus MC MPU to other MPUs focused on adult and geriatric populations, as described in recent (published after the year 2000) publications, it becomes clear that it reflects the broader trend of published MPUs being increasingly situated in academic hospitals [5,17,18,19]. Furthermore, modern MPUs are more frequently integrated into non-psychiatric medical departments [5,13,17,20,21,22], with an emphasis on ensuring access to comprehensive acute medical care [23].

### Strengths and Limitations

This study’s strength lies in its innovative methodology, which departs from a traditional outcome-focused approach by using a theoretical framework grounded in evidence-based practice. Literature emphasizes the need to improve MPU research by thoroughly describing populations and goals [11]. We used consecutive sampling to survey referring physicians, helping to reduce recall bias. Although 29 of 96 physicians did not respond, qualitative data from document analysis and semi-structured interviews provided richer insights into the MPU’s development and implementation—insights often difficult to obtain through surveys alone.

While qualitative methods are influenced by interviewers’ skills and interpretations, we minimized bias by holding multiple consensus meetings (MM, CP, JB) to agree on accurate interpretations of statements and themes. Three negative perceptions of the MPU were noted: (1) a mistaken belief that it was always at full capacity, (2) a perception that MPU nurses needed more medical guidance, and (3) the distance of the MPU from some referring wards. These views may be shaped by the small number of interviewed physicians, most of whom regularly referred to the MPU, potentially introducing positive bias.

Our study captured initial physician expectations but did not assess patient outcomes or post-referral experiences. Nonetheless, such expectations provide valuable insights into how physicians perceive appropriate MPU patients relative to PCS. Though we focused on physician views, we acknowledge the value of including perspectives from nurses, patients, and caregivers. Excluding them is a limitation, and future research should incorporate these voices to fully understand the MPU’s role in integrated care.

Our quantitative analysis used basic univariate statistics without correcting for multiple testing, which requires cautious interpretation aligned with the study’s exploratory intent. A larger sample size could potentially reveal manageability as a more prominent referral factor. Still, our aim was to identify clinically relevant differences, and we do not expect a larger sample would yield significantly new findings. Including behavioral severity or frequency measures might have highlighted additional referral drivers.

Previous research, such as Leue’s, highlights the difficulty of evaluating cost-effectiveness for relatively small-scale interventions [6]. An alternative approach could involve investigating specific elements that contribute to the MPU’s added value, including the perspectives of patients and caregivers [11]. Our findings suggest that MPUs may contribute more to improving care quality, reducing hospital costs, ensuring access for vulnerable groups, and supporting healthcare workers—goals consistent with the quadruple aim— rather than classical societal cost-effectiveness alone. Future studies should consider how MPUs can be designed to align with these broader goals. As noted, different stakeholders prioritize goals differently. This should be explored by including a wider range of perspectives—patients, families, nurses, insurers, and policymakers—and considering how to balance their views. Future research could also identify which MPU elements are universal and which can be tailored to local contexts, factoring in hospital type and PCS collaboration [12].

Patient or caregiver input was not included in this study’s design, as the aim was to evaluate the MPU’s added value from the perspective of referring physicians. We assume referrers consider patient needs and values, but this is an indirect viewpoint. Focusing on referrers is justified here, since our aim was to assess whether the MPU fulfills its founding goals as envisioned by potential users.

This does not imply that patient perspectives lack value. On the contrary, dissatisfaction from patients or informal caregivers would warrant a critical reevaluation of the MPU. However, exploring those experiences would require a separate research aim and design. We are currently planning a qualitative study involving patients and caregivers to understand their experiences across referral wards, transfers, and the MPU itself. This will offer essential insights into the MPU’s value from the perspective of those receiving care.

## Conclusion

In conclusion, our study employed an ‘evidence-based practice’ framework to assess the effectiveness of the Erasmus MC MPU in fulfilling its mission. We addressed the following questions:

**1.** How do physicians, as primary stakeholders, value the MPU?
**2a.** Do referrals align with the intended patient population and do these referrals differ from the PCS population
**2b.** Are there unintended patients groups present and are there unintended and undesirable reasons for referral to the MPU?

The present population of patients is mostly in line with the original motivation for establishing the MPU, and the MPU’s multidisciplinary team, training, and meetings are in accordance with the integrated features one strived for. Regarding the first question, physicians highlighted multiple areas where the MPU has added value, including improved care quality, enhanced job satisfaction, safer practices, strengthened collaboration, reduced stigmatization, and a more vibrant learning environment.

For the second question, the MPU primarily serves patients with disruptive behaviors, such as suicidal tendencies, although these patients are not necessarily unmanageable or aggressive. We did not find undesirable reasons for referral to the MPU.

Regarding unintended referral reasons: disruptive behavior is not a sufficient condition for referral. Instead, referrals are often driven by staff perceptions of their own competencies and the resulting care burden.

We conclude that the MPU at the Erasmus MC was able to live up to expectations due to the integration of medical and psychiatric expertise.

## Additional File

The additional file for this article can be found as follows:

10.5334/ijic.8964.s1 Appendix. Appendix I: The Caarls’ clusters of the Ad Hoc questionnaire. Appendix II: The InDiBI questionnaire. Appendix III: The semi structured interview.

## References

[1] Molnar G, Fava GA, Zielezny MA. Medical-psychiatric unit patients compared with patients in two other services. Psychosomatics. 1985;26(3):193–209. DOI: 10.1016/S0033-3182(85)72874-5 3991860

[2] Stoudemire A, Brown JT, McLeod M, Stewart B, Houpt JL. The combined medical specialties unit: an innovative approach to patient care. N C Med J. 1983;44(6):365–7. 6576231

[3] White EM. The use of a medical support group on a medical/psychiatric unit. Issues in Mental Health Nursing. 1988;9(4):353–62. DOI: 10.3109/01612848809140937 3229982

[4] Fogel BS, Stoudemire A, Houpt JL. Contrasting models for combined medical and psychiatric inpatient treatment. The American journal of psychiatry. 1985;142(9):1085–9. [in eng]. DOI: 10.1176/ajp.142.9.1085 3927752

[5] Dekker L, Heller HM, van der Meij JE, Toor AEJ, Geeraedts LMG. A mixed psychiatric and somatic care unit for trauma patients: 10 years of experience in an urban level I trauma center in the Netherlands. European Journal of Trauma and Emergency Surgery. 2020;46(5):1159–65. DOI: 10.1007/s00068-019-01088-3 30770955

[6] Leue C, Driessen G, Strik JJ, Drukker M, Stockbrügger RW, Kuijpers PM, et al. Managing complex patients on a medical psychiatric unit: An observational study of university hospital costs associated with medical service use, length of stay, and psychiatric intervention. Journal of Psychosomatic Research. 2010;68(3):295–302. DOI: 10.1016/j.jpsychores.2009.04.010 20159217

[7] Kishi Y, Kathol RG. Integrating medical and psychiatric treatment in an inpatient medical setting: The type IV program. Psychosomatics. 1999;40(4):345–55. DOI: 10.1016/S0033-3182(99)71230-2 10402882

[8] Stoudemire A, Kahn M, Brown JT, Linfors E, Houpt JL. Masked depression in a combined medical-psychiatric unit. Psychosomatics. 1985;26(3):221–4, 7–8. DOI: 10.1016/S0033-3182(85)72875-7 3991861

[9] Honig A, Visser I, Heller H, Kieviet N, Boenink AD. Medisch-psychiatrische unit in algemeen ziekenhuis: effectieve gecombineerde somatische en psychiatrische zorg? [A medical-psychiatric unit in a general hospital: effective combined somatic and psychiatric care?]. Ned Tijdschr Geneeskd. 2014;158(5):A6520. [in Dutch] 24472335

[10] Caarls PJ, van Schijndel MA, Kromkamp M, Wierdsma AI, Osse RJ, van der Hoeven G, et al. Need analysis for a new high acuity medical psychiatry unit: which patients are considered for admission? BMC Health Serv Res. 2019;19(1):139. DOI: 10.1186/s12913-019-3967-7 30819164 PMC6394074

[11] Pinto C, Fabbricotti IN, van Wijngaarden J, Hoogendijk WJG, Alsma J, van Busschbach JJ, van Schijndel MA. Moving Beyond the Status Quo of Integrated Inpatient Medical and Psychiatric Care Units: The Path to Real-World Evaluation. Psychiatr Serv. 2022;73(5):555–60. DOI: 10.1176/appi.ps.202000811 34704774

[12] Caarls P, Van Schijndel M, Berk Gvd, Boenink A, Boerman D, Lijmer J, et al. Factors influencing the admission decision for Medical Psychiatry Units: a concept mapping approach. Plos One. 2019;14(9):e0221807. DOI: 10.1371/journal.pone.0221807 31527872 PMC6748432

[13] Maarten A. van Schijndel MDPD, Jeroen DH, van Wijngaarden PD, Joris J, van de Klundert PD. Organization and Outcomes of Integrated Inpatient Medical and Psychiatric Care Units: A Systematic Review. Psychiatric Services. 2022;73(1):64–76. DOI: 10.1176/appi.ps.202000416 34407632

[14] Bachynsky N. Implications for policy: The Triple Aim, Quadruple Aim, and interprofessional collaboration. Nurs Forum. 2020;55(1):54–64. DOI: 10.1111/nuf.12382 31432533

[15] Kathol RG. Cost outcomes on a medical psychiatry unit. Journal of Psychosomatic Research. 2010;68(3):293–4. DOI: 10.1016/j.jpsychores.2009.06.012 20159216

[16] Hussain M, Seitz D. Integrated Models of Care for Medical Inpatients With Psychiatric Disorders: A Systematic Review. Psychosomatics. 2014;55(4):315–25. DOI: 10.1016/j.psym.2013.08.003 24735866

[17] Chan AC, Burke CA, Coffey EM, Hilden DR, Coira DL, Warner-Cohen J, et al. Integrated Inpatient Medical and Psychiatric Care: Experiences of 5 Institutions. Ann Intern Med. 2018;168(11):815–7. DOI: 10.7326/M17-3186 29710091

[18] Alberque C, Gex-Fabry M, Whitaker-Clinch B, Eytan A. The Five-Year Evolution of a Mixed Psychiatric and Somatic Care Unit: A European Experience. Psychosomatics. 2009;50(4):354–61. DOI: 10.1176/appi.psy.50.4.354 19687176

[19] Eytan A, Bovet L, Gex-Fabry M, Alberque C, Ferrero F. Patients’ satisfaction with hospitalization in a mixed psychiatric and somatic care unit. Eur Psychiatry. 2004;19(8):499–501. DOI: 10.1016/j.eurpsy.2004.09.004 15589710

[20] Jansen L, Hunnik F, Busschbach JJV, Lijmer JG. Measuring outcomes on a Medical Psychiatric Unit: HoNOS, CANSAS and costs. Psychiatry Res. 2019;280:112526. DOI: 10.1016/j.psychres.2019.112526 31445422

[21] Jansen LAW, Somanje-Bolweg RRJ, Wierdsma AI, Busschbach JJV, Lijmer JG. Evaluation of opening a type III/IV medical psychiatric unit. Int J Psychiatry Clin Pract. 2021;25(2):147–51. DOI: 10.1080/13651501.2021.1881973 33586580

[22] Wittink MN, Cross W, Goodman J, Jackson H, Lee HB, Olivares T, et al. Taking the Long View in an Inpatient Medical Unit: A Person-Centered, Integrated Team Approach for Patients With Severe Mental Illnesses. Psychiatr Serv. 2020;71(9):885–92. DOI: 10.1176/appi.ps.201900385 32362225

[23] Padrino SL, Chan AC, van Schijndel M, Wittink MN. Medical Psychiatry Units: A Delphi Consensus Approach to Defining Essential Characteristics. J Acad Consult Liaison Psychiatry; 2024. DOI: 10.1016/j.jaclp.2024.09.004 39370111

