# Supplementary material for: A Mixed-method Study on the Implementation of a Medical Psychiatric Care Unit
Source: Int J Integr Care. 2025 Jul 28;25(3):16. doi: 10.5334/ijic.8964 (PMC12315675; doi:10.5334/ijic.8964)
Supplement: Appendix. — Appendix I: The Caarls’ clusters of the Ad Hoc questionnaire. Appendix II: The InDiBI questionnaire. Appendix III: The semi structured interview. [file ijic-25-3-8964-s1.pdf]

I.The Caarls' clusters of the Ad Hoc questionnaire:

Question 1-7 :	Staff competencies	(Cluster 1)
Question 8-15 :	Patient characteristics	(Cluster 2)
Question 16-21 :	Psychiatric symptoms and Behavioral problems	(Cluster 3)
Question 22 :	Medical needs and Capabilities	(Cluster 4)
Question 23 :	Patient Context	(Cluster 5)

Cluster 1: Staff Competencies

1. Preventive, to prevent behavioral and/or psychological problems (Yes/No)
2. There is insufficient expertise on the current department (Yes/No)
3. The nursing care load is too high because of the psychiatric problem (Yes/No)
4. Aftercare is needed for a psychiatric problem (Yes/No)
5. There are problems surrounding the patient's discharge (not being psychiatric aftercare) (Yes/No)
6. There is a shortage of beds on the regular ward (Yes/No)
7. The problem is too complicated for treatment elsewhere (Yes/No)

Cluster 2: Patient Characteristics

8. A psychiatric problem is prominent (Yes/No)
9. Further diagnostics are needed to determine the cause of the behavior (psychological or somatic) (Yes/No)
10. There is a psychiatric history (Yes/No)
11. There is a history of substance abuse (Yes/No)
12. A psychiatric or behavioral disorder interferes with medical treatment (Yes/No)
13. Treatment on the regular ward is impeded by the psychiatric or behavioral problem or vice versa (Yes/No)
14. Sufficient recovery is expected within the foreseeable future for transfer to a psychiatric or somatic ward, elsewhere or home (Yes/No)
15. The psychiatric or behavioral problem is the result of a medical treatment or medical condition (Yes/No)

Cluster 3: Psychiatric Symptoms and Behavioral Problems

16. There is a personality disorder (Yes/No)
17. There is an intoxication or substance abuse issue (Yes/No)
18. Delirium is present (Yes/No)
19. The patient has committed a suicide attempt (Yes/No)
20. There is evidence of suicidality (Yes/No)
21. There is aggression towards others (Yes/No)

Cluster 4: Medical Needs and Capabilities

22. There is a too complicated medical problem in a psychiatric patient (Yes/No)

Cluster 5: Patient Context

23. There is a problematic interaction between patient and medical staff (Yes/No)

II.InDiBI questionnaire

Inpatient Disruptive Behavior Index 5.0 (InDiBI 5.0)
Patient indentification:

Instruction: Indicate presence of disruptive behavior and whether the patient is manageable. Consider the most serious behaviors that you have experienced with the patient during the current admission.

Definition of disruptive behavior: Behavior of the patient that you find irritating and/or annoying.

Definition of manageability: If the patient's behavior does not obstruct the progress of nursing care, diagnostics and/or treatment, the patient is manageable.

1. Is there disruptive behavior by the patient?
   a. ☐ Patient shows no disruptive behavior and is manageable
   b. ☐ Patient exhibits disruptive behavior, but is manageable
   c. ☐ Patient exhibits disruptive behavior and is not manageable
2. What type of disruptive behavior does the patient display? Several answers are possible.
   a. ☐ Aggression
   b. ☐ Suicidal behavior
   c. ☐ Self-harm
   d. ☐ Claiming behavior
   e. ☐ Uncooperative
   f. ☐ Delusions
   g. ☐ Hallucinations
   h. ☐ Agitation
   i. ☐ Disinhibition or loss of decorum
   j. ☐ Shouting and screaming
   k. ☐ Wandering
   l. ☐ Inertia
   m. ☐ Apathy
   n. ☐ Refusal to eat and/or drink

III.Semi structured interview

Introduction:
What is an MPU?
What are the capabilities of an MPU?
What kind of patients do you think are treated there?
What do you think should be possible at an MPU?
Why is an MPU needed in addition to the consultative service?

Added value of an MPU
What do you think is the added value of the MPU here at Erasmus MC?
What do you think about the establishment of an MPU here at the EMC since January 2019? In other words, do you think there is a need for an MPU?
Do you think the MPU makes an important contribution in providing good care for complex patients?
What do you think are the advantages and disadvantages of an MPU?
If so, what do you think are the advantages and disadvantages compared to the psychiatric consultative service?
What do you think of the MPU's response to a referral?
Why did you choose to refer the patient to the MPU and not the consultative service? Or vice versa.
Was there no alternative? Such as rooming-in (family member), or transfer to the mental health system.

Added value in suicide care?
What do you think about patients being admitted to the MPU as a result of a suicide attempt?
Do you think the MPU is important for the care of suicide attempt patients?
How do you feel about having psychiatric patients on your own ward?

Logistics?
How do you find the organization of the MPU? Does the referral of a patient go smoothly?
When a patient is referred, can they get to the MPU right away?

Finance
Are there any financial barriers?
Do you think the MPU will come out positive if a cost-benefit analysis will be done?

Evaluation:
What do you think are the strengths of the MPU?
What do you think are the non-strong points of the MPU?
Are there any specific success stories?

Other:
Do you know anyone else who often refers to the MPU? If so, would you please email his/her contact information?
Do you know anyone else who never refers to the MPU and has suitable patients for it? If so, would you please email his/her contact information?
Do you have any additional comments?
